# Can uterine artery embolization be an alternative to plastic and reconstructive uterus operation by minimally invasive surgery?

**DOI:** 10.3205/iprs000157

**Published:** 2021-06-09

**Authors:** Cristina Cezar, Luz Angela Torres de la Roche, Jörg Hennefründ, Hugo Christian Verhoeven, Rajesh Devassy, Rudy Leon De Wilde, Working Group on Minimally Invasive Therapy in Benign Disease of the Uterine Wall (European Society of Gynecological Endoscopy, ESGE)

**Affiliations:** 1 University Hospital for Gynecology, Pius Hospital, Carl von Ossietzky University Oldenburg, Germany; 2 Tagesklinik Oldenburg, Germany; 3 Private Center for Endocrinology, Preventive Medicine, Reproductive Medicine and Gynecology, Dusseldorf, Germany

**Keywords:** uterine artery embolization, myoma, complications, gynaecologic surgical procedures, haemostatic techniques

## Abstract

**Introduction:** Plastic and reconstructive minimally invasive surgery has been established as gold standard in myomectomy. Therapy failure eventually leads to future surgical interventions or hysterectomy: surgeons and patients should be aware of the risks and benefits. We conducted a systematic review to analyse the evidence on the therapeutic indications and adverse events associated with uterine artery embolization and thereby evaluating if this method could be a valid alternative therapy.

**Methods:** In concordance with PRISMA guidelines, literature research was made in PubMed, Cochrane Library, UpToDate, Amboss and Medline databases. Clinical trials, reviews and case reports published in English between January 2010 and June 2020 were included.

**Results:** 44 articles were included out of 838 papers identified at initial search. Regarding uterine fibroids, three original papers and one Cochrane review reported the benefits of the procedure as an alternative to surgery, even in large and giant fibroids. Furthermore, several studies discussed the use of embolization for postpartum haemorrhage to decrease rates of hysterectomy after other haemostatic methods were exhausted, because of the potential risk of abnormal placentation in a future pregnancy. The procedure can also be successfully used as prophylactic method in different obstetrical procedures.

**Conclusions:** The use of embolization in different uterine pathologies is a minimally invasive procedure as an alternative to surgery, especially in women who desire to preserve their uterus. Its related complications are described and can be avoided by a stringent indication of the procedure. More evidence regarding fertility after UAE, use of the procedure prophylactically in obstetrical haemorrhage or in adenomyosis is needed.

## Zusammenfassung

**Einleitung:** Die plastische und rekonstruktive minimal-invasive Chirurgie hat sich als Goldstandard in der Myomektomie etabliert. Therapieversagen führt schließlich zu weiteren chirurgischen Eingriffen oder zur Hysterektomie: Chirurgen und Patientinnen sollten sich der Risiken und des Nutzens bewusst sein. Wir führten eine systematische Übersichtsarbeit durch, um die Evidenz zu den therapeutischen Indikationen und unerwünschten Ereignissen im Zusammenhang mit der Uterusarterienembolisation zu analysieren und damit zu bewerten, ob diese Methode eine valide Therapiealternative darstellen könnte.

**Methoden:** In Übereinstimmung mit dem PRISMA Leitfaden recherchierten wir die medizinische Literatur in den Datenbanken PubMed, Cochrane Library, UpToDate, Amboss und Medline. Klinische Studien, Reviews und Case Reports, die zwischen Januar 2010 und Juni 2020 in Englisch veröffentlicht wurden, waren einbezogen.

**Ergebnisse:** Von den 838 bei der ersten Suche identifizierten Arbeiten wurden 44 Artikel eingeschlossen. In Bezug auf Uterusmyome berichteten drei Originalarbeiten und ein Cochrane-Review über die Vorteile des Verfahrens als Alternative zur Operation, auch bei großen und riesigen Myomen. Darüber hinaus wurde in mehreren Studien der Einsatz der Embolisation bei postpartalen Blutungen diskutiert, um die Rate der Hysterektomie zu senken, nachdem andere hämostatische Methoden wegen des potentiellen Risikos einer abnormalen Plazentation in einer zukünftigen Schwangerschaft ausgeschöpft waren. Das Verfahren kann auch erfolgreich als prophylaktische Methode bei verschiedenen geburtshilflichen Eingriffen eingesetzt werden.

**Schlussfolgerungen:** Die Anwendung der Embolisation bei verschiedenen uterinen Pathologien ist ein minimal-invasives Verfahren als Alternative zur Operation, insbesondere bei Frauen, die ihren Uterus erhalten möchten. Die damit verbundenen Komplikationen sind beschrieben und können durch eine stringente Indikationsstellung des Verfahrens vermieden werden. Mehr Evidenz bezüglich der Fertilität nach UAE, des prophylaktischen Einsatzes des Verfahrens bei geburtshilflichen Blutungen oder bei Adenomyose ist erforderlich.

## Introduction

Gold standard of care in myomectomy is the plastic and reconstructive minimally invasive uterine surgery [1]. The benefits of uterine artery embolization (UAE) in the treatment of different uterine pathologies are demonstrated for women desiring organ preservation, being minimally invasive, even in the treatment of giant fibroids [2]. Since its introduction in 1995 as conservative therapy for fibroids [3], the intervention has been developed, being applied for uterine pathologies such as myoma [4], postpartum haemorrhage (PPH) [5], or as a prophylactic procedure before obstetrical surgery with a high risk of massive bleeding [6], adenomyosis [7], and uterine vascular malformation [8]. However complications are possible [9], [10], which may increase the likelihood of therapy failure eventually leading to future surgical interventions [11].

## Material and methods

This review was performed according to the PRISMA guidelines [12]. In addition to clinical trials, we also analysed several case reports and case series in order to collect as much information as possible regarding the whole spectrum of complications. The research was performed using PubMed, Cochrane Library, UpToDate, Amboss and Medline databases, limited to articles published between January 2010 and June 2020. The search term combination consisted of words and words variations for “uterine artery embolization”, “indications”, “major complications” and “minor complications”. Only articles published in English were selected. Exclusion criteria were: associated pathology as risk factors for venous thrombosis such as postpartum period, bed rest, blood transfusion, pelvic varicose veins and inherited thrombophilia, studies focused on technical aspects, earlier studies – EMMY trial 2006, FIBROID 2005, 2008, REST 2007 and also other minimally invasive procedure such as LUAO (laparoscopic uterine artery occlusion) – MARA 2012 study. Letters and editorials were also excluded. 

## Results

**Figure 1:**
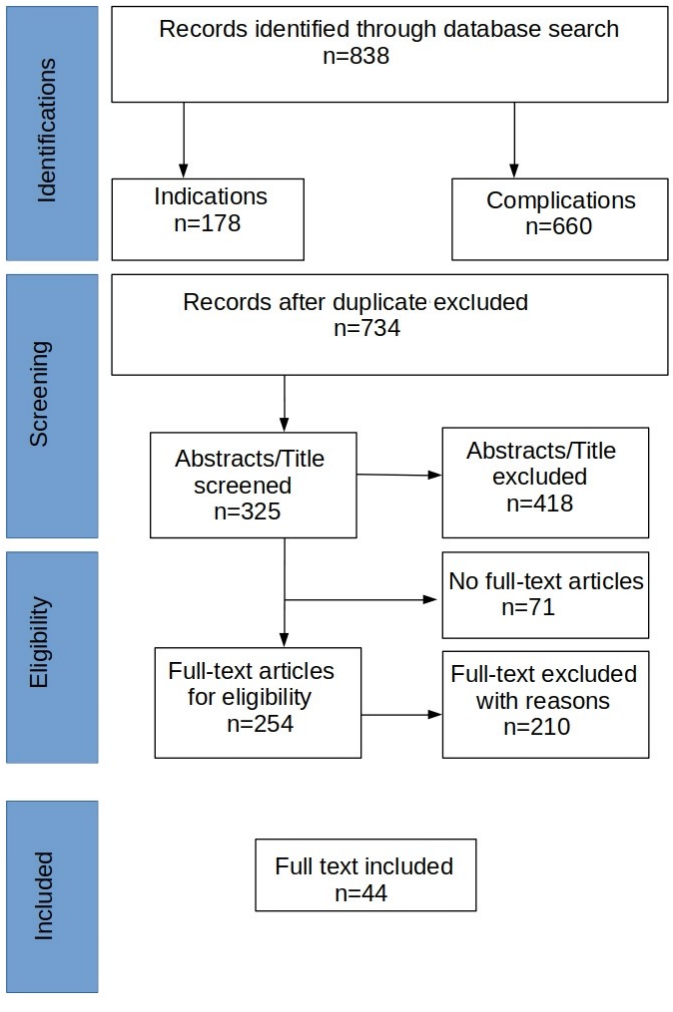
PRISMA flow chart of the reports selection

Our initial database search identified a total of 838 records (n=178 for indications and n=660 for complications). After the duplicates were removed, 734 records remained and were further screened independently by the first two authors. The screening excluded 418 papers which were irrelevant to the topic of our review, discussing other aspects beside indications and outcomes of UAE. 71 further records were excluded, due to no full text availability. Out of 254 eligible articles, 210 were excluded according to our exclusion criteria: a total of 44 articles were eligible and included in this review (Figure 1 (Fig. 1)). 

### Indications of UAE

#### Uterine fibroids

Most of the studies included in our review discussed the use of UAE as an important tool in the treatment of myomas. The procedure proved to be an effective therapy for uterine fibroids in women with heavy menstrual bleeding who were resistant to other conservative therapies [13] and proposed as an alternative myoma therapy in women wishing to preserve fertility. However, the outcomes on fertility are still unclear [14]. 

The procedure can be performed irrespective of the number of fibroids and of their uterine location (submucosal, intramural or subserosal) [4]. Pedunculated fibroids are a relative contraindication of UAE, due to the risk of degeneration and subsequent infection which may lead to more or less severe complications [15], [16]. 

Three original papers REST 2011 [11], FUME 2012 [17], EMMY 2016 [18], and one Cochrane review studied the effects of UAE versus surgery, either myomectomy or hysterectomy, which we included (Table 1 (Tab. 1)). 

The REST 2011 study (Randomised comparison of uterine artery embolization with surgical treatment in patients with symptomatic uterine fibroids) compared the results of UAE versus surgery (myomectomy and hysterectomy) in 157 women with symptomatic fibroids. The study was multicentric, used a randomization 2:1 (106 UAE and 51 surgery) and a long follow-up of 5 years [11]. The improvement in quality of life was similar in both groups, with most complications occurring in the first 12 months; the authors concluded that the safety profile of UAE is equivalent to that of surgery. Even if the rate of reintervention was higher in the UAE group, it remained an alternative to surgery [11]. 

The FUME 2012 study (Fibroids of the Uterus: Myomectomy versus Embolization) compared the quality of life in patients following UAE or myomectomy performed for uterine fibroids in a prospective cohort [17]. The outcomes during a follow-up period of 5 years showed similar results regarding significant improvement in quality of life, but myomectomy remained more robust as procedure with fewer reinterventions and complications [17]. 

The EMMY 2016 study (Embolization vs. Hysterectomy) was a multicentric randomized controlled trial conducted between 2002 and 2004 which included 177 patients with symptomatic uterine fibroids who were treated either conservatively, UAE, or operatively by hysterectomy [18]. The study population was randomized to 81 UAE and 75 hysterectomies with the goal of comparing the outcomes and quality of life 10 years after the procedure [18], [19]. The authors concluded that every woman with symptomatic fibroids could be counselled about UAE as an alternative to hysterectomy, given that in 69% of cases a hysterectomy was avoided, and the satisfaction rates did not differ in both groups [18]. 

According to a Cochrane Review in 2014 [20], UAE remains a good alternative for the treatment of uterine fibroids, compared to surgical methods like myomectomy or hysterectomy, as it is associated with a shorter hospital stay and quicker recovery and is also rarely associated with the need of blood transfusion [20]. However it was observed that UAE was associated with more complications, but a direct correlation to major complications was inconclusive [20].

#### UAE to improve fertility

Regarding the use of UAE to improve fertility in women with fibroids, Karlsen et al. [21] analysed the results of 17 studies including 989 patients. The authors observed that in women receiving UAE the rates of pregnancy were lower and the rates of miscarriage were higher, when compared to those with myomectomy. However, they also discussed the low quality of evidence, indicating the necessity of further studies to elucidate this aspect [21] (Table 2 (Tab. 2)). 

The effects of UAE on ovarian reserve is a subject of interest for clinicians and the results from different studies seem to indicate a negative influence of the procedure on ovarian reserve, however, the authors underlined the necessity of further studies to verify these outcomes [8]. The age of the patients plays an important role, with younger women having a decreased risk of ovarian failure after UAE [22]. Another approach to improve fertility in women with large fibroids could be the combination of UAE followed by myomectomy [23], [24]. 

#### UAE in large and very large fibroids 

Regarding the size of the fibroids to be treated, previous trials have demonstrated a low evidence (level 1) [2] in the use of UAE as treatment of most fibroid sizes [11], [18]. 

The effectiveness and safety of UAE in the treatment of large and very large fibroids have been debated [2]: we found one retrospective study [25] and four case reports [26], [27], [28], [29] (Table 3 (Tab. 3)) indicating a higher risk of complications such as uterine necrosis and infection, possible leading to sepsis and necessity of hysterectomy, or even life threatening situations due to ischemia of adjacent organs such as toxic megacolon or perforation of transverse colon [26]. 

Regarding the issue of large fibroids, a new report discussed the possibility of the aberrant vascularization of large fibroids from neighbouring abdominal structures, a situation which requires a better consideration of cases to be embolized [30]. In this report, 24 cases of large fibroids treated between 2008 and 2017 were analysed. In all subjects, arteriography and embolization were performed prior to subsequent myomectomy or total hysterectomy. In three cases the arteriography showed the aberrant vascularization of the large fibroids from omental arteries and gastroepiploic arteries [30]. However, this kind of complications are rare [31], [30]. Concerning the safety and effectiveness of UAE in fibroids larger than 10 cm, Bérczi et al. reported the results of 303 patients underlying no differences observed between fibroids smaller and larger than 10 cm [29].

#### UAE prophylactic use in obstetrical procedures and postpartum haemorrhage

A further useful indication of UAE is as a prophylactic method before obstetrical procedures with high risk of massive bleeding such as dilatation and curettage associated with uterine ectopic pregnancy (cervical or caesarean scar) [6], termination of pregnancy with complete placenta praevia (CPP) [32], [33], suspected gestational trophoblastic disease, or retained placenta with vascularity [5], [6] (Table 4 (Tab. 4)). 

Concerning the effects on future pregnancy, different authors reported that UAE might induce endometrial ischemia and necrosis, potentially followed by abnormal placentation, with afterwards affected uteroplacental exchange and possible intrauterine growth restriction [23], placenta accrete [34], [35], placenta increta [36], and placenta praevia [37]. The leading factors which contribute to necrosis are reduced blood flow as well as damage to the endomyometrium [38]. 

Other previous studies and trials have documented that several procedures to control the bleeding such as uterine compression sutures (B-Lynch) or Bakri balloon tamponade have to be taken into consideration by clinicians before proceeding to embolization, as these haemostatic methods seem not to be associated with increased risk of abnormal placentation [39], [40], [41], [42]. 

#### UAE for adenomyosis

Concerning the use of UAE, a recent review reports a good level of evidence regarding the acceptance and safety of the procedure, but the evidence is low regarding fertility outcomes after the procedure [7], [43].

### Complications of the UAE

In defining the complications of UAE several criteria and classifications are used [4], [11], [23], [28]. According to SIR (Society of Interventional Radiology), the complications of UAE can be divided in five grades [11], the first two categories being considered as minor and the other three as major complications.

1. No therapy, no consequence.
2. No therapy, no consequence, but including overnight observation.
3. Therapy needed, short hospitalization <48 hours.
4. Major therapy needed, prolonged hospitalisation >48 hours.
5. Permanent sequelae for the patient [44].

Depending on the moment of onset, the complications can be divided into acute, subacute and chronic [4]:

1. Acute (in the first 24 hours after UAE): such as PES – post embolization syndrome, septic bleeding, pulmonary embolus, vasovagal response [45].
2. Subacute (up to one week) and chronic: urinary retention, local infections such as endometritis or abscesses, amenorrhea, necrosis, fibroid expulsion [13].

Classifications regarding the intensity of the events:

1. Minor complications [28]

- Post-embolization syndrome (PES) induced by an inflammatory response to the necrotic tissue and characterized by fever, nausea and vomiting, local pain and leukocytosis [46].
- Fibroid passage [47] after UAE with severe menstrual cramping, vaginal discharge, sometimes fibroid expulsion or even severe bleeding [28].

2. Major complications [23]

- Events related to technical procedure: iodine allergy, vessel dissection or aneurysm, arterial thrombosis or pulmonary embolism.
- Ischemic necrotic complications which could be limited to genital area or at distance due to the passage of embolizing particles into vessels.
- Infection: puncture site infections, up to severe forms such as provoked endometritis and sepsis.
- Death by large pulmonary embolus or sepsis with multiorgan system failure, due to uterine necrosis.

Another important aspect to be taken into account is the risk of repeated UAE and reintervention. According to the REST trial [11], the rates of reinterventions were 13% at 1 year to 32% at 5 years, compared to 2% in organ saving surgery at 1 and 5 years [47]. 

UAE complications’ incidence:

- Prolonged vaginal discharge 2–17% [47]
- Discharge with fever 4% [47]
- UAE failure 4% [47]
- Urinary stress incontinence 3.7%, pressure symptoms 2.8% or menorrhagia 2.6% [47]
- Postembolization syndrome 2.86% [47]
- Fibroid expulsion 1–10% [48], [49], [50], [51] ranging up to 50% for submucosal fibroids [52]. A cervical passage of fibroids can occur even 3 years after the procedure has been done [53]. The submucosal and intracavitary location of the fibroids is more frequently associated with the expulsion which could lead to a septic complication [28]. Therefore, preprocedural MR was proposed to localize the myoma and thereby improve the outcomes of the intervention performed on selected patients [28].
- Septicaemia 1–3% [47]
- Pulmonary embolization <1% or deep vein thrombosis <1% [47]
- Rare complications at the urinary bladder as vesical fissure <1% or intra-abdominal abscess <1% [47]
- Permanent amenorrhoea 0–3% under 45 years and 20–40% in older women [54]
- Less common complications such as infectious events and even septic reactions due to the material used or injuries of nerves and vessels [48]
- Patients requiring hysterectomy due to the complications <1% [48]

## Discussion

The present paper summarizes the current publications regarding the indications and complications of UAE in different uterine pathologies. Despite the large heterogeneity of the reports and reviews presented, there is a trend to consider UAE as an alternative therapy, as the major complications are rare, sustained by the previous metaanalysis [10]. Several papers described serious and severe complications especially when UAE was performed in large fibroids [2], [26], [27], [28], [29]; these complications may be avoided by stringent indication of the intervention after preprocedural imaging such as MRI or even CT [30], [47]. 

As an approach to treat postpartum haemorrhage, UAE has been used as a prophylactic method in high-risk patients before different abortive procedures to avoid massive bleeding, to decrease rates of hysterectomy [6]. Because of the risk of subsequent placenta accreta in future pregnancies, clinicians should consider standard methods to stop massive bleeding before proceeding to embolization [39], [40], [41], [42]. Cases should be made on an individual basis and discussed with the patient before establishing a therapeutic plan as shared consent. 

The strength of this work is the extensive incorporation of research published since 2010, including case reports and case series up to clinical trials and studies. A limitation of this review is that only articles published in English language were taken into consideration, eventually excluding other relevant articles reported in other languages. In addition, the heterogeneity in the definition of major and minor complications within studies limited the qualitative analysis. 

Despite the limitations, the actual trend useful for the clinicians as much as for the patients is to consider UAE as a tool to treat women with different uterine pathologies. UAE showed acceptable clinical outcomes in the treatment of uterine myomata, even in the management of fibroids larger than 10 cm. However, due to the risk of possible complications, imaging such as MR or CT is advised to be performed before the procedure. Complications are possible, so these problems should be discussed with the patient before establishing a therapeutic plan.

## Conclusion

Current data show that UAE is an acceptable procedure to treat different uterine pathologies. However, several trials and studies discuss the high risk of complications and reinterventions, emphasizing the importance of proper selection of women that could benefit from the procedure. Patients with various symptomatic uterine pathologies can be counselled about the possibility of UAE as an alternative to surgery such as myomectomy or hysterectomy. 

Regarding future research, several aspects have to be clarified such as fertility after UAE, the use of the procedure in obstetrical haemorrhage, in adenomyosis, and eventually comparing it with other minimally invasive treatment by means of high frequency ultrasound using newly designed prospective randomized controlled studies.

## Notes

### Competing interests

The authors declare that they have no competing interests.

### Authors’ contributions 

RDW, CC and LATR: conceptualization. CC and LATR: data curation, formal analysis, writing original study report. All authors read, corrected and approved the final manuscript, based on their expertise.

### Acknowledgments 

Jennifer Eidswick for language corrections. 

## Figures and Tables

**Table 1:**
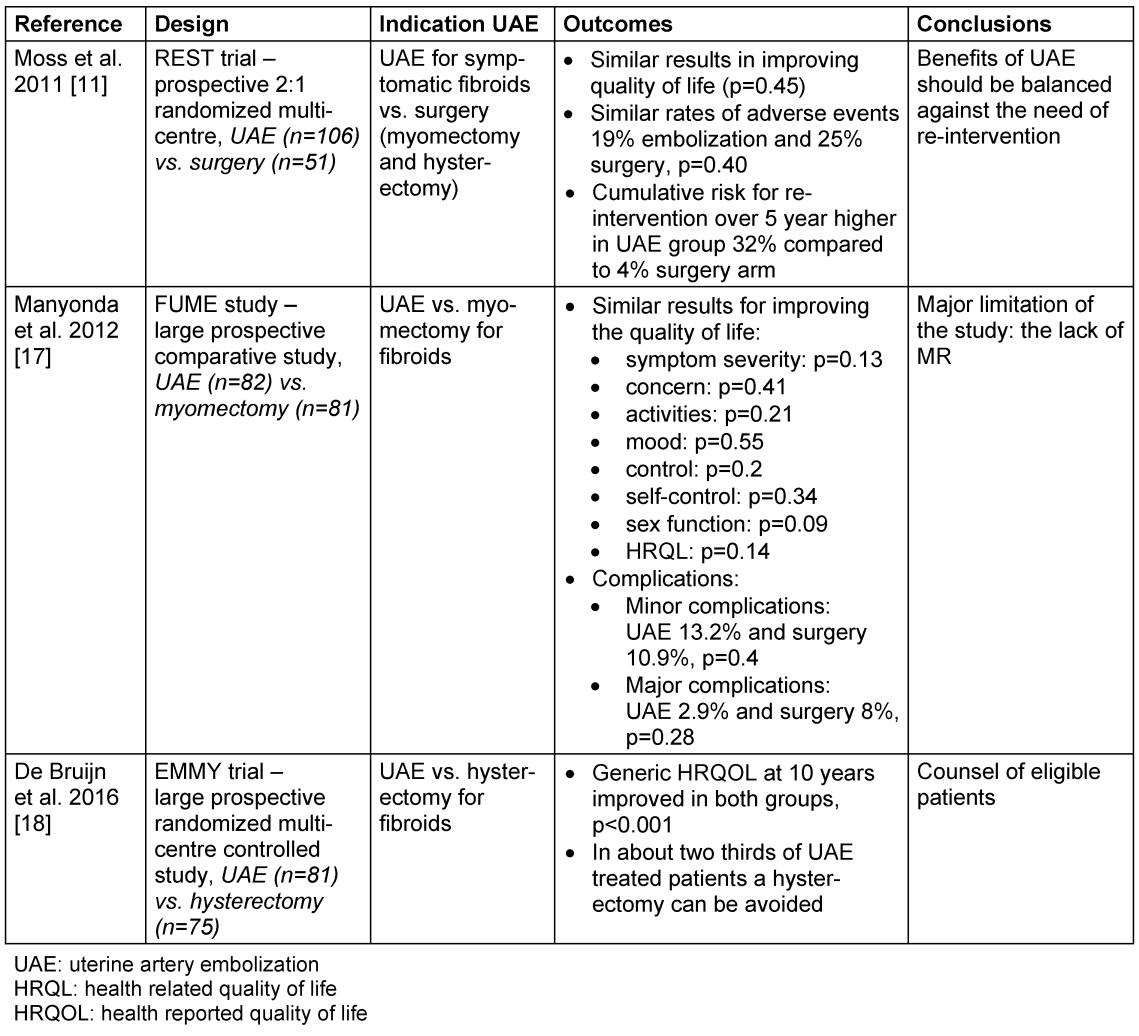
Uterine artery embolization (UAE) versus surgery (myomectomy and/or hysterectomy) in the treatment of fibroids

**Table 2:**
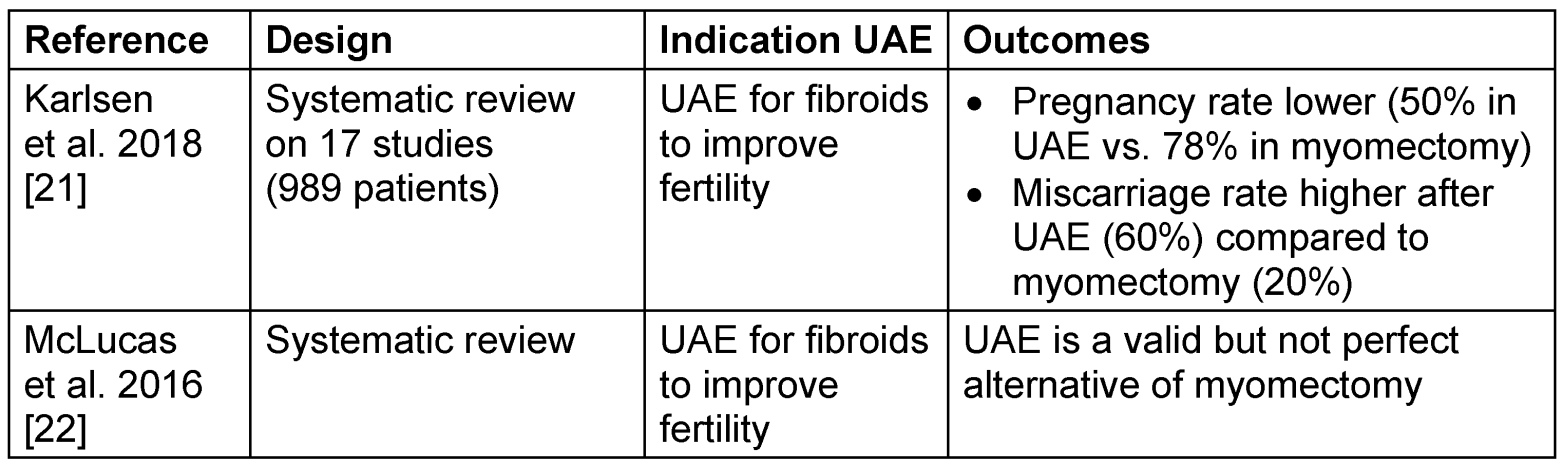
Fertility after uterine artery embolization for fibroids

**Table 3:**
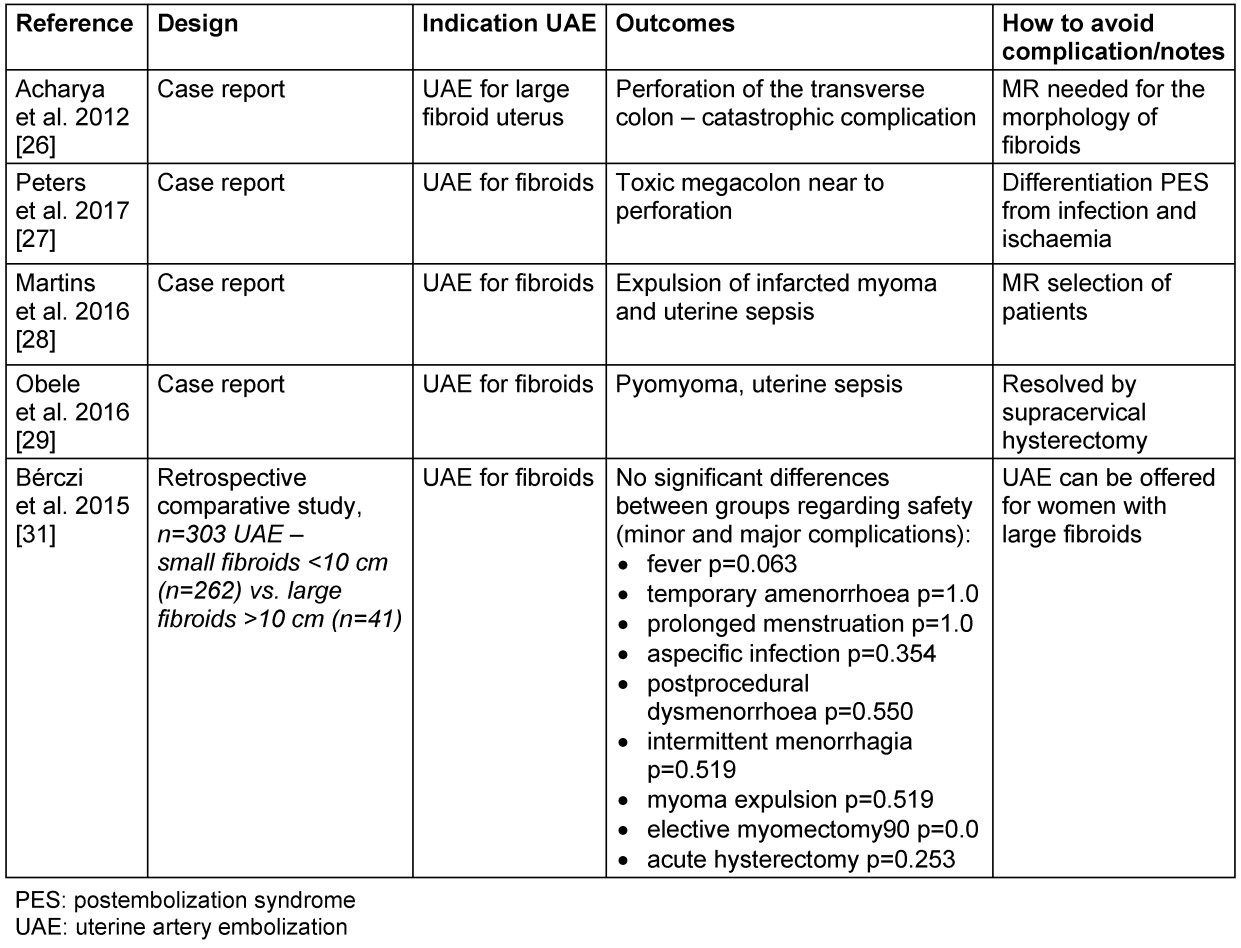
Uterine artery embolization for large and very large fibroids

**Table 4:**
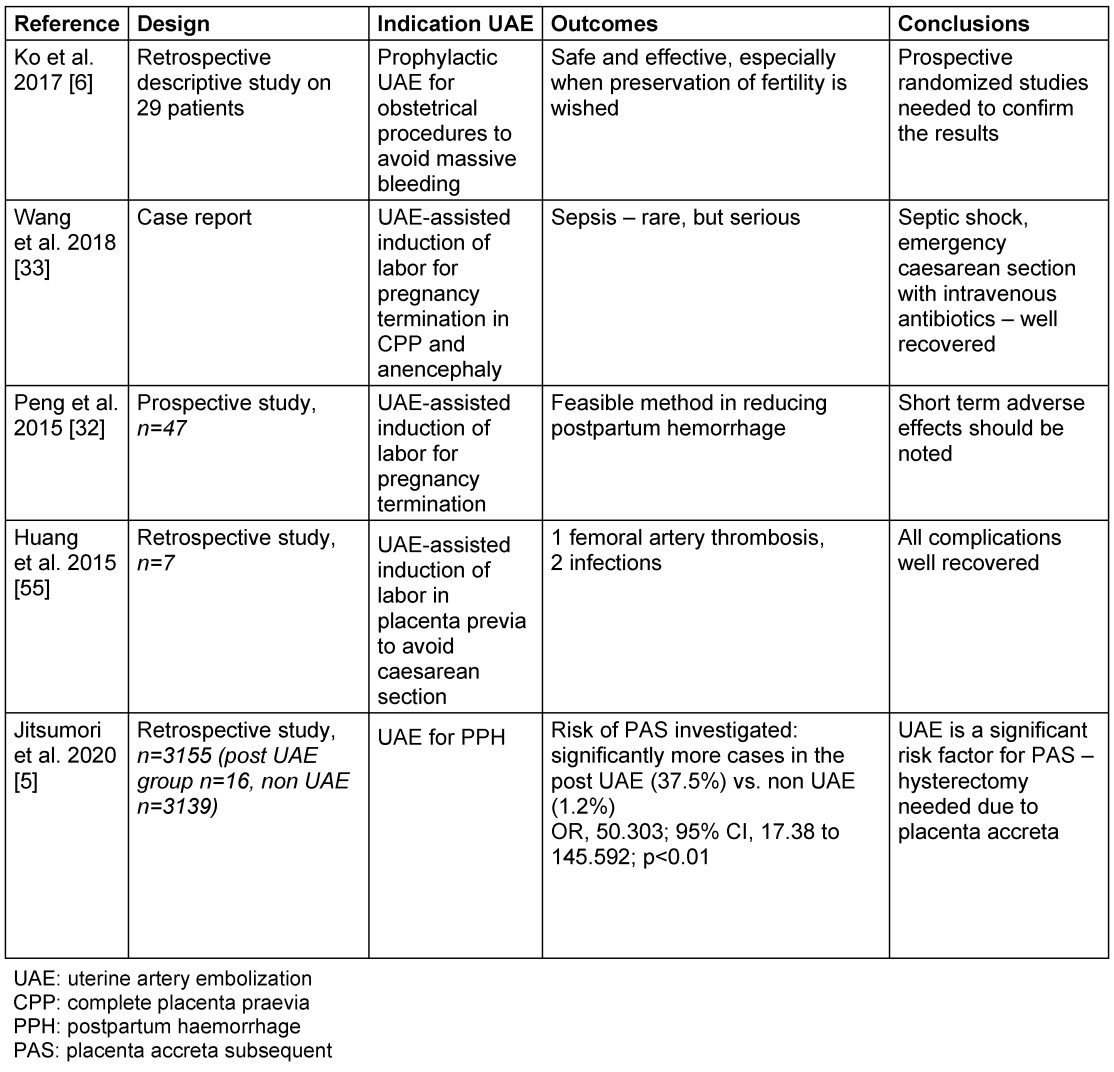
Uterine artery embolization before obstetrical procedures and for postpartum haemorrhage
